# Marine Compounds with Anti-*Candida* sp. Activity: A Promised “Land” for New Antifungals

**DOI:** 10.3390/jof8070669

**Published:** 2022-06-25

**Authors:** Anelise Maria Costa Vasconcelos Alves, Natália Cruz-Martins, Célia Fortuna Rodrigues

**Affiliations:** 1 Institute of Health Sciences, University of International Integration of Afro-Brazilian Lusophony, Av. da Abolição, 3-Centro, Redenção 62790-000, Ceará, Brazil; anelisealves@unilab.edu.br; 2 LEPABE—Laboratory for Process Engineering, Environment, Biotechnology and Energy, Faculty of Engineering, University of Porto, Rua Dr. Roberto Frias, 4200-465 Porto, Portugal; 3 ALiCE—Associate Laboratory in Chemical Engineering, Faculty of Engineering, University of Porto, Rua Dr. Roberto Frias, 4200-465 Porto, Portugal; 4 TOXRUN—Toxicology Research Unit, Cooperativa de Ensino Superior Politécnico e Universitário—CESPU, 4585-116 Gandra PRD, Portugal; ncmartins@med.up.pt; 5 Institute for Research and Innovation in Health (i3S), University of Porto, Rua Alfredo Allen, 4200-135 Porto, Portugal

**Keywords:** *Candida albicans*, *Candida*, marine compound, sea, polymer, drug, resistance, bioactivity, pathogen

## Abstract

*Candida albicans* is still the major yeast causing human fungal infections. Nevertheless, in the last decades, non-*Candida albicans Candida* species (NCACs) (e.g., *Candida glabrata*, *Candida tropicalis*, and *Candida parapsilosis*) have been increasingly linked to *Candida* sp. infections, mainly in immunocompromised and hospitalized patients. The escalade of antifungal resistance among *Candida* sp. demands broadly effective and cost-efficient therapeutic strategies to treat candidiasis. Marine environments have shown to be a rich source of a plethora of natural compounds with substantial antimicrobial bioactivities, even against resistant pathogens, such as *Candida* sp. This short review intends to briefly summarize the most recent marine compounds that have evidenced anti-*Candida* sp. activity. Here, we show that the number of compounds discovered in the last years with antifungal activity is growing. These drugs have a good potential to be used for the treatment of candidiasis, but disappointedly the reports have devoted a high focus on *C. albicans*, neglecting the NCACs, highlighting the need to perform outspreading studies in the near future.

## 1. Introduction

*Candida albicans* is the most highly adapted human fungal pathogen causing yeast infections. However, in the last decade, the non-*Candida* *albicans Candida* species (NCACs) (e.g., *Candida glabrata*, *Candida krusei*, *Candida tropicalis*, or *Candida parapsilosis*) have been escalating due to the widespread use and even overuse of broad-spectrum antimicrobial drugs. In addition, and more alarmingly, NCACs have been linked to high rates of antifungal resistance and chronic infections [1,2,3].

Microbial infectious diseases are a serious global health problem. Candidiasis is one of the most predominant fungal infections, particularly in critically ill patients, such as immunosuppressed individuals, under prolonged use of broad-spectrum antibiotics, with HIV, cancer, and even the elderly [4,5,6,7,8]. The growth of antimicrobial resistance (AMR) among both bacteria and fungi is still escalating, and, by 2050, about 10 million people are expected to die per year, and billions will be spent [8,9]. Up-to-date reports indicate that the increase in AMR also involves *Candida* sp. [2,10,11,12], and mounting evidence has supported the major role of other diseases (e.g., cardiovascular, neurological) in this interplay [13,14]. It is, thus, imperative to slow down the rise of AMR (in *Candida* sp.), to cut the demand for antifungal drugs, and to increase the supply of new and effective drugs against drug-resistant microorganisms [15]. Henceforward, promoting the investment in alternative antifungal drugs and refining the existing ones is of global urgency to extend the current stock of drugs. In this sense, the search for novel anti-*Candida* sp. drugs is imperative, particularly from natural compounds, such as those deriving from marine sources.

Oceans and deep seas cover approximately 70% of the Earth and are the habitat of around 80% of all species [16,17], reaching bacteria, fungi, invertebrates, and complex organisms [18]. In the 1950s, Bergmann et al. discovered the first marine compounds with pharmacological importance (antivirals)—spongothymidine and spongouridine—extracted from *Tectitethya crypta* (Caribbean sponge) [19]. In the last years, the advances in technological approaches (e.g., remotely operated vehicles or closed-circuit computerized mixed gas rebreathers) have made it possible to deeply explore the marine environment in order to search for novel compounds [19,20]. More than 5000 novel natural products have been extracted from marine organisms living in sea environments [21,22]. Conversely, marine compounds are a source of powerful drugs with antifungal (and/or antibiofilm) potential [23,24,25,26,27]. In fact, marine environments are a huge pool of natural compounds with biological importance, besides being safe for human use as antifungals [23,24]. This may be an incomparable chance for the search and formulation of new drugs [23], particularly in human biomedical applications [28]. Marine biopolymers are also presently an active research area towards a deeper knowledge of effective drug delivery systems and to develop novel therapies. These compounds are highly interesting as biomaterials for clinical applications due to their abundance, biocompatibility, biodegradability, ease of surface modification, inexpensiveness, stability, and non-toxic nature [24].

In this sense, this brief review recapitulates the marine compounds that have been recently studied owing to their anti-*Candida* effects. The search was performed using the NBCI platform (PubMed), using the keywords “anti-candida”, ”marine”, ”drugs”, and selecting the years 2017 to 2022.

## 2. Marine Compounds, Potential Antimicrobial Activity, and Current Drug Marine Biotech

Natural compounds are one of the best alternative resources to explore bioactive drug candidates. Undeniably, these compounds have properties that have proved to be beneficial for the management of candidiasis, also eliminating and/or out-competing with the involved microorganisms. In addition, they have been explored in biotech research and industry, proving to be interesting alternatives to the currently available biopolymers. The next sections will discuss these points.

### Marine Compounds with Anti-Candida sp. Effects

There is an array of marine compounds that have been identified as having antimicrobial activity. Recently, the isolation of certain components of mollusks’ defense systems has been studied. Hong Kong oysters (*Crassostrea hongkongensis*) infected by *Vibrio parahaemolyticus* produce peptides with potent antibacterial and antifungal activity, showing biological activity against *C. albicans*, and without cytotoxicity in vitro and in rodents [29]. Turbinmicin, a compound from a sea squirt microbiome constituent, *Micromonospora* sp., has evidenced to be a potent antifungal activity in vitro and in vivo against *Candida auris*. In addition, it was shown to be a safe drug, targeting the Sec14 of the vesicular trafficking pathway [30]. Similarly, the American oyster (*Crassostrea virginica*) showed to produce a defensive compound exhibiting strong antimicrobial activity. Seo and colleagues [31] designed peptides arginine-rich analogs into antibiotic candidates (A0, A1, A2, A3, and A4), the last two showed action against *C. albicans* growth without displaying cell toxicity [31]. More complex organism extracts, such as the Fish Skin Mucous (FSM) derived from *Dasyatis pastinaca*, have also shown significant and species-specific activity against strains of *Candida* sp. It is thought that these results may be linked to a chitinase expression, which targets chitin, a structural polysaccharide found in large proportions in the fungal wall [32]. The Q-Griffithsin (Q-GRFT), a derivation of a marine red algal lectin, also binds to the cell wall and exhibits activity against strains of *Candida* sp. Its mechanism of action is through to be related to the induction of cell death by disruption of cell wall integrity after binding to α-mannan, and the trigger of reactive oxidative species (ROS) formation. Moreover, the Q-GRFT inhibits the growth of *C. glabrata*, *C. parapsilosis*, and *C. krusei* and *C. auris* [33].

However, not only does red algae exert anti-*Candida* effects, but the crude extracts of seaweeds also obtained from the Riacho Doce beach, Alagoas (Brazil), present minimum inhibitory concentrations (MIC), ranging from 0.03 to 16.00 μg/mL against *Trichophyton rubrum*, *Trichophyton tonsurans*, *Trichophyton mentagrophytes*, *Microsporum canis*, *Microsporum gypseum*, and yeasts *C. albicans*, *C. krusei*, *Candida guilliermondi*, and *C. parapsilosis*, respectively. When the extraction methods were compared, the dichloromethane, methanol, and ethanol extracts showed the largest inhibition abilities against *C. albicans* growth [34]. Peptides and proteins that have shown anti-*Candida* properties are summarized in Table 1.

Additionally, from several sponge species, a large arsenal of other molecules can be isolated: nucleosides, terpenes, sterols, cyclic peptides, and alkaloids (Table 2), presenting huge pharmacological importance. The EtOH extract of *Oceanapia* sp., a marine sponge, contains Oceanalin B, a sphingoid tetrahydoisoquinoline β-glycosides, with remarkable in vitro activity against *C. glabrata* [37]. In addition to molecules produced directly by sponges, it is possible to obtain products from microorganisms living in symbiosis with them. The symbiotic organisms are bacteria, archaea, microalgae, and fungi, totaling a volume of 40% of the sponge’s body. These microorganism communities hosted in sponges produce bioactive secondary metabolites with antimicrobial effects. Recently, culturing fungi under different conditions has resulted in the production of new secondary metabolites that are not observed previously in regular culture protocols. Two dimeric antifungal alkaloids, entitled fusarypyridines A and B, were isolated from the organic extract of the fungus *Fusarium* sp. LY019 living in symbiosis with the sponge *Suberea mollis* in the Red Sea. These compounds showed selective and potent effects against *C. albicans* (MIC values of 8.0 and 8.0 µM, respectively) [38]. Evidence suggests the alkaloid derivatives 6a and 6b antifungal have activity against *C. albicans*, *C. glabrata*, *C. tropicalis*, and *C. krusei*. A synergistic in vitro effect was observed with ketoconazole by binding to membrane ergosterol and, consequently, triggering the lysis of fungal cells [39]. Similarly, the sponge *Callyspongia siphonella* hosts the actinomycete strain *Streptomyces coelicolor* LY001. The extract obtained from this strain was purified and gave rise to chlorinated derivatives of 3-phenylpropanoic acid (*n* = 3) that demonstrated significant and selective activities against *C. albicans* [40]. In another study, new non-brominated pyrrole-2-carboxamide alkaloids, nakamurins A–C (1–3) were obtained from the sponge *Agelas nakamurai* (Xisha Islands, South China Sea). Compound 2 was identified as an antimicrobial with weak activity against *C. albicans* (MIC = 60 mg/mL) and no toxic effect in vitro [41]. In addition, *Hemimycale* sp., a species endemic to the Red Sea, produces C–E hemimycalins (secondary metabolites) that inhibit the growth of bacteria and fungi such as *Escherichia coli* and *C. albicans* [42].

Other marine chemical compounds have shown effective antifungal effects (Table 3, Table 4 and Table 5. Oceanapiside (OPS), a sphingolipid, presents a fungicidal effect against fluconazole-resistant *C. glabrata*. Probably, the inhibition of polarized growth promoted by OPS is due to the disruption of organized actin. Phytosphingosine (PHS) was able to reverse OPS activity which indicates that it blocks fungal sphingolipid metabolism by specifically inhibiting the conversion step of PHS to phytoceramide [58]. Similarly, marine polyunsaturated fatty acids inhibit biofilm formation of *C. albicans* and *Candida dubliniensis*. Mitochondrial metabolism and biofilm biomass are affected by these fatty acids, as well as the cell wall morphology [59].

The Antarctic emperor penguin (*Aptenodytes forsteri*) also hosts bacteria that produce antimicrobial secondary metabolites. For example, the NJES-13T, an actinobacterial strain present in its gut microbiota, is a producer of anguciclinone, gepyromycin (GPM), and 2-hydroxy-tetrangomycin (2-HT), as well as complex bioflocculation active exopolysaccharide (EPS) metabolites. It was observed the angucycline/angucyclinone derivative metabolites have been isolated from this strain with inhibitory activity against the microorganisms *Staphylococcus aureus*, *Bacillus subtilis*, and *C. albicans* [44]. Similarly, some strains of *Aspergillus* sp. are antimicrobial producers. Among the antimicrobial compounds from *Aspergillus* species are observed xanthones, alkaloids, cyclic peptides, and terpenes. Wu et al. (2020) studied the extract of *Aspergillus terreus* RA2905 hosted in the sea hare *Aplysia pulmonica*. In this extract, they isolated two new thiodiketopiperazines, emestrins L and M, five analogs (3–7), and five dihydroisocoumarins (8–12). Among the isolated compounds, only the number 3 presented activity against *C. albicans* ATCC10231 (MIC 32 μg/mL) [57]. However, eight compounds isolated from an *Aspergillus fumigatus* D strain, living in symbiosis with the species *Edgeworthia chrysantha Lindl*, showed no significant effect on *C. albicans*. The *Penicillium* genus may also produce bioactive compounds as immunosuppressants, harmful mycotoxins, antibacterial and antifungal. The extract of *Penicillium* sp. SY2107 from Mariana Trench sediment prepared in a rice medium inhibits microbial growth [46]. In addition, another team characterized the antimicrobial activity of organic extracts of fungi belonging to the genera *Penicillium*, *Cladosporium*, *Emericellopsis*, and *Plectosphaerella*. The study observed 49 mycotoxins and functional metabolites, indicating a great chemical diversity. The highest number of compounds was isolated from fungi of the genus *Penicillium*, on average 165 parent ions per strain. Regarding bioactivity, *Penicillium* sp. 31.68F1B was the strain with the most expressive results. This fungus showed activity against seven plant and human pathogens, and *C. albicans* was the microorganism most sensitive to the compounds of this strain [47]. Likewise, eutypellenoid B, isolated from the extract of *Eutypella* sp. D-1 inhibits fungi growth of the following species *C. parapsilosis* (MIC= 8 μg/mL), *C. albicans* (MIC= 8 μg/mL), *C. glabrata* (MIC =16 μg/mL), and *C. tropicalis* (MIC= 32 μg/mL) [48].

Marine bacteria are also targets for pharmacotherapeutic studies. For example, new compounds belonging to the alkaloid class were recently isolated from an *Acinetobacter* sp. ZZ1275 strain. These compounds are Indolepyrazines A and B, and they showed antimicrobial activities against methicillin-resistant *S. aureus*, *E. coli*, and *C. albicans* with MIC values of 12 μg/mL, 8–10 μg/mL, and 12–14 μg/mL, respectively [45]. Another interesting finding is the synthetic molecules based on natural compounds, such as the N-methylated analog proline-rich tetracyclopeptide that presented efficient antifungal effects against *C. albicans* [35]. The *Candida* sp. biofilms treated with purified marine bacterial DNase (MBD) from a strain of *Vibrio alginolyticus* (AMSII) were also capable of decreasing 60–80% biomass and significantly reducing the rate of biofilms formation on urinary catheters while disrupts yeast to dimorphic hyphae switch in *C. albicans* [36].

On the other hand, some compounds from marine-derived microorganisms have also shown to be inefficient as antifungals. The calcomycins isolated from the in vitro culture of a strain of *Streptomyces* sp. HK-2006-1 shows selective activity against only bacteria such as *S. aureus* while not affecting the growth of fungi such as *C. albicans* and *Aspergillus niger* [56]. These and other works that have studied specific marine compounds for anti-*Candida* sp. activity are summarized in Table 1, Table 2, Table 3, Table 4 and Table 5.

## 3. Final Remarks

AMR has hugely complicated the treatment of infections while facilitating the progression to chronic or recurrent stages, and thus new antimicrobial drugs are urgently necessary for effective antibiotic therapy. Specifically, the search for new antimicrobials within the species that inhabit the oceans seems promissory, leading to the identification of a series of novel compounds with a great ability to manage fungal infections, including candidiasis.

Marine environments are an almost inexhaustible source of resources of varied natural compounds, with substantial antimicrobial bioactivities, even against resistant pathogens [71]. Among the marine taxa, bacteria, fungi themselves, sponges, and soft corals, have shown to be a rich source (but not only) of novel biologically active drugs. In addition, the use of marine animals and microorganisms has the advantage of having low associated costs and a great yield to produce anti-*Candida* sp. drugs. Regrettably, we noticed a strong gap within the *Candida* sp. that have been tested in the published reports. In fact, almost only exclusively *C. albicans* has been the target of an intense study, setting aside, in almost all studies, the NCACs. Considering that these *Candida* sp. are emerging as strong human pathogens in the last decades and have been linked to high rates of antifungal resistance (such as *C. glabrata* and *Candida auris*) [72], it is, thus, of utmost interest to extend these studies to the remaining (fungal) species.

## Figures and Tables

**Table 1:** Antifungal activity of marine peptides and proteins.

Compound	Origin	*Candida* sp.	Reference
Up-regulated peptides (URPs) from Oyster plasma	Oyster (*Crassostrea hongkongensis*) infected by *V. parahaemolyticus*	*Candida albicans*	[29]
American oyster defensin (AOD)- arginine-rich analogs (A3 and A4)	Oyster *Crassostrea virginica*	*Candida albicans*	[31]
N-methylated proline-rich tetrapeptides	*Pseudomonas* sp. and *Pseudoalteromonas* sp.	*Candida albicans*	[35]
Peptide URP20	*Crassostrea hongkongensis*	*Candida albicans*	[29]
Marine bacterial DNase (MBD)	*Vibrio alginolyticus*	*Candida albicans*	[36]
Q-Griffithsin (Q-GRFT)	Marine red algae	*Candida albicans*, *Candida glabrata*, *Candida parapsilosis*, *Candida krusei*, *Candida auris*	[33]

**Table 2:** Antifungal activity of marine alkaloids and esters.

Compound	Origin	*Candida* sp.	Reference
Emethacin C	*Aspergillus terreus* RA2905	*Candida albicans*	[43]
C-2 hydroxyl substitutes; 2-hydroxy-tetrangomycin; 2-hydroxy-frigocyclinone	Strain NJES-13^T^	*Candida albicans*	[44]
Indolepyrazines A and B	*Acinetobacter* sp. ZZ1275	*Candida albicans*	[45]
Andrastones B and C	*Penicillium* sp. SY2107	*Candida albicans*	[46]
Fusaripyridines A and B	*Fusarium* sp. LY019	*Candida albicans*	[38]
31.68F1B	*Penicillium* sp.	*Candida albicans*	[47]
Diketopiperazine alkaloids cyclo(l-Phe-*trans*-4-OH-l-Pro) and Cyclo(l-Phe-*cis*-4-OH-d-Pro)	*Streptomyces coelicolor* LY001 from the sponge *Callyspongia siphonella*	*Candida albicans*	[40]
Eutypellenoid B	*Eutypella* sp. D-1	*Candida parapsilosis*, *Candida albicans*, *Candida glabrata*, *Candida tropicalis*	[48]
Talaromanloid A, Talaromydene, 10-hydroxy-8-demethyltalaromydine, 11-hydroxy-8-demethyltalaromydine, talaromylectone, and ditalaromylectones A and B	*Talaromyces mangshanicus* BTBU20211089	*Candida albicans*	[49]
Rubrofusarin B, Alternariol 9-*O*-methyl ether, Fonsecinone D, Asperpyrone A, Asperpyrone D, Fonsecinone B, Fonsecinone A, and Aurasperone A	*Aspergillus fumigatus* D	*Candida albicans*	[50]
*Asperfuranone*; *Asperpyranone A and B*	*Aspergillus terreus* RA2905	*Candida albicans*	[43]
Aspergillusidone G; Aspergillusidone F; Aspergillusidone C; 2-Chlorounguinol and Aspergillusidone A	*Aspergillus unguis*	*Candida albicans*	[51]
Isoagelasidine B, Isoagelasine C and diterpene alkaloids	Sponge *Agelas nakamurai*	*Candida albicans*	[41]
Ascochlorin/Fimetarin analogues	*Stilbella fimetaria*	*Candida albicans*	[52]
Isocoumarin analogues	*Paraphoma* sp. CUGBMF180003	*Candida albicans*	[53]
Diketopiperazines	*Aspergillus versicolor* MF180151	*Candida albicans*	[54]
Piperazine-triones	Deep-sea-derived *Streptomycetes* sp. strain SMS636	*Candida albicans*	[55]
Chalcomycins	*Streptomyces* sp. HK-2006-1	*Candida albicans*	[56]
Hemimycalins C-E	Red Sea Marine Sponge *Hemimycale* sp.	*Candida albicans*	[42]
Thiodiketopiperazine and 3,4-Dihydroisocoumarin Derivatives	Marine-Derived Fungus *Aspergillus terreus*	*Candida albicans*	[57]
Turbinmicin	Sea squirt	*Candida auris*	[30]
Alkaloids 6a and 6 b	Marine sponge	*Candida albicans*, *Candida glabrata*, *Candida tropicalis*, *Candida krusei*	[39]

**Table 3:** Antifungal activity of marine extracts and sub-products.

Compound	Origin	*Candida* sp.	Reference
*Arthrospira platensis* extracts by Supercritical carbon dioxide extraction (SFE-CO_2_)	*Arthrospira platensis*	*Candida albicans* ATCC 10231	[60]
Pure fractions from MEF 134	Marine endophytic fungi (MEF) 134	*Candida albicans*	[61]
Extracts from *Corallimorphus* cf. *pilatus* and *Stomphia coccinea*	Deep-Sea Anemones (*Corallimorphus* cf. *pilatus* and *Stomphia coccinea*)	*Candida albicans*	[62]
Algae crude extracts	Marine macroalgae (*Ulva lactuca Linnaeus*, *Padina gymnospora*, *Sargassum vulgare*, *Hypnea musciform*, *Digenea simplex*)	*Candida albicans*, *Candida krusei*, *Candida guilliermondi*, *Candida parapsilosis*	[34]
CyanoCoating	Cyanobacterium *Crocosphaera chwakensis* CCY0110	*Candida albicans*	[63]
Fish Skin Mucous (FSM)	*Dasyatis pastinaca*	*Candida albicans* ATCC90028, *Candida albicans* ATCC10231, *Candida albicans* clinical strain 6, *Candida albicans* clinical strain 10, *Candida glabrata* clinical strain 14, *Candida tropicalis* clinical strain 21	[32]

**Table 4:** Antifungal activity of fatty acids and lipids of marine organisms.

Compound	Origin	*Candida* sp.	Reference
Pentadecanoic acidPentadecanal	Synthetic origin	*Candida albicans*	[64]
3-O-β-D-galactopyranosyl-1-O-3,6,9,12,15-octadecapentaenoyl-2-O-tetradecanoylglycerol 1 (MGDG)	*Karenia mikimotoi*	*Candida albicans*	[65]
(2S)-3-O-β-D-galactopyranosyl-1-O-3,6,9,12,15-octadecapentaenoylglycerol 2 (MGMG)	*Karenia mikimotoi*	*Candida albicans*	[65]
Methyl (3Z,6Z,9Z,12Z,15Z)-octadeca-3,6,9,12,15-pentaenoate 3 (PUFAME)	*Karenia mikimotoi*	*Candida albicans*	[65]
Sesquiterpenoids and Steroids	Coral *Sinularia brassica*	*Candida albicans*	[66]
Oceanalin B	Sponge *Oceanapia* sp.	*Candida glabrata*	[37]
Marine Polyunsaturated Fatty Acids	Synthetic origin	*Candida albicans* and *Candida dubliniensis*	[59]
Oceanapiside (OPS)	Marine sponge *Oceanapia* sp.	*Candida glabrata*	[58]

**Table 5:** Antifungal activity of marine carbohydrates.

Compound	Origin	*Candida* sp.	Reference
Bengazole A	*Jaspis cf coriacea*	*Candida albicans*	[67]
Isocoumarin analogues	*Paraphoma* sp. CUGBMF180003	*Candida albicans*	[53]
Exopolysaccharide	*Porphyridium marinum*	*Candida albicans*	[68]
Agarose oligosaccharide	Marine red algae	*Candida albicans*	[69]
Alginate-chitosan oligosaccharide	Ocean algae	*Candida albicans*	[70]
